# Resolvin D1 Reduces Lung Infection and Inflammation Activating Resolution in Cystic Fibrosis

**DOI:** 10.3389/fimmu.2020.00581

**Published:** 2020-04-28

**Authors:** Elisa Isopi, Domenico Mattoscio, Marilina Codagnone, Veronica Cecilia Mari, Alessia Lamolinara, Sara Patruno, Marco D’Aurora, Eleonora Cianci, Annalisa Nespoli, Sara Franchi, Valentina Gatta, Marc Dubourdeau, Paolo Moretti, Maria Di Sabatino, Manuela Iezzi, Mario Romano, Antonio Recchiuti

**Affiliations:** ^1^Center for Advanced Studies and Technology, Department of Medical, Oral and Biotechnology Science, “G. d’Annunzio” University of Chieti – Pescara, Chieti, Italy; ^2^Center for Advanced Studies and Technology, Department of Medicine and Aging Sciences, “G. d’Annunzio” University of Chieti – Pescara, Chieti, Italy; ^3^Center for Advanced Studies and Technology, Department of Psychological, Humanistic and Territorial Sciences, “G. d’Annunzio” University of Chieti – Pescara, Chieti, Italy; ^4^Ambiotis SAS, Toulouse, France; ^5^Cystic Fibrosis Regional Center, Ospedale “San Liberatore,” Atri, Italy

**Keywords:** pro-resolving lipid mediators (SPM), inflammation, resolution, macrophages, neutrophils, infection, *Pseudomonas aeruginosa*

## Abstract

Non-resolving lung inflammation and *Pseudomonas aeruginosa* infections are the underlying cause of morbidity and mortality in cystic fibrosis (CF). The endogenous lipid mediator resolvin (Rv) D1 is a potent regulator of resolution, and its roles, actions, and therapeutic potential in CF are of interest. Here, we investigated actions and efficacy of RvD1 in preclinical models of cystic fibrosis. *Cftr* knockout mice with chronic *P. aeruginosa* lung infection were treated with RvD1 to assess differences in lung bacterial load, inflammation, and tissue damage. Cells from volunteers with CF were treated with RvD1 during *ex vivo* infection with *P. aeruginosa*, and effects on phagocytosis and inflammatory signaling were determined. In CF mice, RvD1 reduced bacterial burden, neutrophil infiltration, and histological signs of lung pathology, improving clinical scores of diseases. Mechanistically, RvD1 increased macrophage-mediated bacterial and leukocyte clearance *in vivo*. The clinical significance of these findings is supported by actions in primary leukocytes and epithelial cells from volunteers with CF where RvD1 enhanced *P. aeruginosa* phagocytosis and reduced genes and proteins associated to NF-κB activation and leukocyte infiltration. Concentration of RvD1 in sputum from patients with CF was also inversely correlated to those of cytokines and chemokines involved in CF lung pathology. These findings demonstrate efficacy of RvD1 in enhancing resolution of lung inflammation and infections and provide proof of concept for its potential as a prototypic novel pro-resolutive therapeutic approach for CF.

## Introduction

Resolution, the ideal outcome of inflammation, is an active process regulated by a superfamily of autacoids termed specialized pro-resolving lipid mediators (SPM) that reduce inflammation without compromising immune defenses, protect from tissue damage, and augment removal of microbes and apoptotic leukocytes, allowing the return to homeostasis (1, 2). Resolvin (Rv) D1 is an SPM that proved organ protective activities and therapeutic potential in sepsis, pneumonia, acute lung injury, sickle cell disease, and asthma (3–7). *In vivo* and *in vitro*, RvD1 reduces polymorphonuclear neutrophil (PMN) interactions with endothelial cells (8) and chemotaxis (9), thus limiting excessive PMN infiltration into inflammatory loci without altering their host protective functions (10). It also counters inflammatory mediators (10) and enhances phagocytosis by macrophages (MΦ) (11), shifting their phenotypes and curbing inflammatory signaling (12).

Non-resolving inflammation and persistent infections are underlying mechanisms of progressive lung disease and premature deaths of patients with cystic fibrosis (CF) (13). Inflammation in CF begins and becomes persistent soon after birth even in the absence of detectable infections, is exaggerated relative to the degree of bacterial burden, impairs host defenses, and destroys airway architecture (14–17). It is hallmarked by an unrelenting infiltration of PMN and high concentrations of inflammatory mediators that perpetuate this tissue destructive response (18, 19) and hyper-responsiveness of MΦ and airway epithelial cells to dangerous signals (20–22).

Continuous antibiotic therapies, along with advances in diagnosis, care delivery, and nutritional support, have improved life quality and median life expectancy of patients to ∼40 years. Despite this, the emergence of multi-drug resistant species, mainly *Pseudomonas aeruginosa*, renders the management of patients extremely challenging (23).

The defective CF transmembrane conductance regulator (CFTR) function, with related mucus stasis and impaired mucociliary clearance, has important roles in the initiation and propagation of airway inflammation in patients with CF (13, 24). With the introduction of correctors and potentiators, individuals with some mutations are recovering CFTR function. While some CFTR modulators reduce the number of pulmonary exacerbations and of patients requiring i.v. antibiotic prescription in the short term (25, 26), other studies indicate that bacterial titer and concentrations of inflammatory mediators in the airways remain elevated in patients even after prolonged treatment with modulators (27–29), indicating that the impact of these therapies on lung inflammation and infection is unclear. Therefore, new approaches to control excessive lung inflammation, enhance bacterial clearance, and improve resolution to alleviate the disease burden are urgently needed.

Accruing evidence indicates that resolution mechanisms are defective in CF. The pro-resolving protein annexin A1 is downregulated in nasal epithelial cells (30), and concentrations of lipoxin A4 are reduced in bronchoalveolar lavage fluid (BALF) of patients with CF (31, 32), possibly because of a CFTR-mediated reduction in their biosynthesis (33), and ratios of sputum concentrations of RvD1 to interleukin (IL)-8 are diminished in individuals with CF (34). Furthermore, CF MΦ and epithelial cells have a significantly lower expression of ALX/FPR2, a RvD1 receptor, which results in impaired antimicrobial and pro-resolutive responses of these cells (35, 36). Overall, these abnormalities can contribute to CF lung inflammation and infection. In addition, recent data indicate that RvD1 improves ion transport and normalizes airway surface liquid height that can improve mucociliary clearance in human bronchial epithelial cells from donors with CF (37). Based on these findings, new therapeutic approaches based on the restoration of SPM biosynthesis are currently being tested in clinical trials (38, 39). Thus, actions of SPM in CF are of interest and may offer basis for innovative pro-resolutive therapeutics to reduce the burden of inflammation-driven lung pathology in patients.

In the present study, we hypothesized that RvD1 could activate resolution in CF and investigated its efficacy and mechanisms of action in preclinical models of disease. Herein, we report that RvD1 treatment enhances resolution of *P. aeruginosa* infection and inflammation in CF mice, and stimulates microbial clearance by human cells while dampening inflammatory signaling that contributes to the excessive inflammation in CF lungs.

## Materials and Methods

### Chemicals and Cell Culture Reagents

RvD1-free acid was purchased from Cayman Chemical (Ann Arbor, MI, United States) and used as previously reported (3, 12). Cell culture media and growth supplements were from Gibco (Thermo Fisher Scientific, Carlsbad, CA, United States) unless otherwise indicated. Media and agar for bacterial growth were from Liofilchem (Roseto degli Abruzzi, Italy).

### Study Participants, Sample Collection, and Analyses

Adult (*n* = 11, > 18 years of age) volunteers with confirmed diagnosis of CF, infected with *P. aeruginosa*, with FEV1 >40% of predicted values, and no history of pulmonary exacerbations in the 4 weeks prior to entering the study [as defined by Fuchs et al. (40)] were recruited at the Regional CF Center of Atri (TE, Italy). Routine care and treatments, including physiotherapy, oral antibiotics, pancreatic enzymes, and CFTR modulators, prescribed by the CF care physicians were continued throughout the study. Four volunteers (two females) had pulmonary exacerbations during the study; sputa were not collected in the following 4 weeks. Volunteers without CF (*n* = 8, > 18 years of age) were enrolled as controls. Sputum was collected upon spontaneous expectoration, processed, and stored within 2 h, as recommended by the CFF Therapeutics Development Network Coordinating Center (41). RvD1 and cytokines/chemokines were measured using a competitive ELISA method or a Luminex multi-analyte assay (ProcartaPlex, Thermo Fisher Scientific, Monza, Italy). Sputa were homogenized on ice and diluted 9- to 10-fold in ultra-pure water (for RvD1) or Dulbecco’s phosphate-buffered saline (DPBS) (for protein assays).

### RP73 Growth and Mice Chronic Infection

The clinical strain of *P. aeruginosa* RP73 (kindly provided by B. Tümmler, Medizinische Hochschule Hannover, Germany), isolated at the late stage of chronic infection from a patient with CF, was used for *in vivo* and *in vitro* experiments as in ref. (3). For *in vivo* chronic infection, RP73 was grown in tryptic soy broth (TSB) to mid-log phase (OD_600__nm_ = 0.45 ± 0.05; ∼ 2 × 10^8^ CFU/mL) and 16 OD (∼ 50 mL) were included into 100- to 200-μm diameter tryptic soy agar (TSA) beads that were inoculated intra-tracheally (i.t.) within 24 to 48 h [see ref. (3)].

Male and female *Cftr* KO [B6.129P2-Cftr^TM 1*UNC*^TgN(FABPCFTR)] mice and WT littermates (42) were obtained from the Cystic Fibrosis animal Core Facility (CFaCore), husbanded in semi-barrier cages, and fed *ad libitum* tap water and chow pellet diet (25/18 CR, Mucedola s.r.l. Settimo Milanese, Italy). Diet contained ∼4% fats as a mixture of palmitic (C16:0, 5.0 g/kg), stearic (18:0, 0.8 g/kg), palmitoleic (ω-7 16:1, 0.3 g/kg), oleic (ω-9 18:1, 4.7 g/kg), linoleic (ω-6 18:2, 11.7 g/kg), and linolenic acid (ω-6 18:3, 1.2 g/kg). Mice (8–12 weeks) were infected i.t. with agar-embedded RP73 (∼ 3.5 × 10^6^ CFU/mouse) for short- and long-term period (5 and 21 days, respectively). RvD1 (100 ng/mouse) or equal amount of vehicle (0.5% _*vol/vol*_ EtOH) were administered via intragastric gavage of 0.2 mL of saline starting at 1-day post-infection (DPI, then daily) or at 5 DPI (then 3 times/week). Mice were monitored daily for clinical signs of disease, and those that lost ≥20% body weight or showed evidence of severe clinical disease were euthanized before the termination of the experiment.

### BALF and Lung Analyses

BALF was collected from mice by injecting three aliquots of sterile DPBS i.t. (1 mL each) aspirated with a 22G (0.9 × 25 mm) catheter connected to a 1 mL syringe. Total leukocytes present in BALF were counted using Turk’s solution and stained (15 min, 4°C) with 0.2 μg/5 × 10^5^ cells of fluorochrome-tagged antibodies (all from Biolegend) against the following antigens: CD16/32 (clone 93), Ter-119 CD45 (Clone 30-F11), CD11b (clone M1/7), Ly6C (clone HK1.4), F4/80 (clone BM8), Ly6G (clone 1A8), CD3ε (clone 145-2c-11). Samples were analyzed with a FACS Canto II flow cytometer (Becton Dickinson, Milan) and the FACS Diva (BD Bioscience) or FCS Express 6 (*DeNovo* Software, Glendale, CA, United States) software.

Viable RP73 cells in BALF and aseptically dissociated lungs were determined upon serial dilutions (10^–1^ down to 10^–6^), plating on TSA, and overnight growth at 37°C. Cytokines and chemokines were measured with Luminex (Millipore, Vimodrone, Italy) multiplex arrays.

For liquid chromatography-tandem mass spectrometry (LC-MS/MS)-based lipidomics, lungs were rapidly dissociated in ice and snap frozen (at −80°C) to prevent further degradation of lipid mediators. The extraction protocol and analysis of bioactive lipids were performed as described in Le Faouder, Baillif et al. (43) and adapted by the Ambiotis SAS (Toulouse, France) standard operating procedures. Samples were taken to solid phase extraction in the presence of deuterated internal standards, and lipid mediators (LM) were eluted in HCOOMe. After solvent evaporation, samples were dissolved in MeOH and injected into an Agilent 1290 Infinity high-performance liquid chromatography (HPLC) system equipped with a Kinetex Biphenyl column (2.1 mm, 50 mm, 1.8 μm) (Phenomenex). LM were eluted with a binary gradient of water/formic acid 0.1% and acetonitrile/formic acid 0.1% and taken to MS/MS analysis on a triple quadrupole Agilent 6490 instrument. LM were identified based on matching of retention time to authentic standards. Calibration curves were obtained using authentic LM mixtures, and quantification was carried out based on peak areas from multiple reaction monitoring (MRM) transitions (43).

For histopathology, mouse lungs excised *en bloc* were inflated with 1 mL of DPBS to permit even organ expansion (critical for quantitative morphometry), fixed, and cut transversally to the trachea into 5.2 mm-thick, parallel slabs/lung, starting from the top 2 mm of the lung to ensure uniform random sampling. Slabs were embedded and cut surface-down into 2-μm sections that were stained with H/E (BioOptica, Milan) to detect inflammatory cell infiltrates. Semi-quantitative scores of lung pathology were assigned on a 0–3 scale, based on criteria described in the Supplementary Table 1 and in ref. (3).

### Primary CF Cell Culture, Infection, and Gene Microarray

CF bronchial epithelial cells (CFBEC) were isolated from bronchi of patients with the ΔF508/ΔF508 genotype and provided by Dr. L. Galietta and collaborators (Istituto G. Gaslini, Genoa, Italy) as part of the Primary Culture Service of the Fondazione Ricerca Fibrosi Cistica (Verona, Italy). CFBEC were grown on rat tail collagen-coated cell culture Petri dishes in serum-free medium (LHC9:RPMI 1640, 1:1) with growth factors as in ref. (44). For further differentiation, CFBEC (2.5 × 10^6^ cells) were grown in air liquid interface (ALI) conditions on 24-mm Transwell filters (0.4 μm pore Ø) for 8–10 days in differentiation medium (Ham’s F12, 2% Ultroser G) (Pall Corp., New York) to obtain tight, fully developed, pseudo-stratified epithelia as previously reported using this protocol (44).

MΦ were differentiated from peripheral blood monocytes isolated as in ref. (11) from de-identified study participants and seeded at 0.5–1 × 10^6^/plate in 6-well plates 24–72 h prior to experiments.

Before infection, CFBEC and MΦ were treated with RvD1 (10 nM) or vehicle for 15 min at 37°C. The infection was established by adding RP73 (∼7.5 × 10^6^ CFU/plate), grown as above, to cells in antibiotic free medium. Medium was removed after 3 h, cells were washed twice, and RNA-isolated using the Macherey-Nagel (Düren, Germany) kit or the Quick-RNA kit from Zymo Research (Irvine, CA, United States). Total RNA extracted from CFBEC and MΦ cells was linearly amplified, labeled with Cy3/5, and hybridized on HOA_007 Human Whole Genome OneArray Microarray V7 (29,264 probes; Phalanx Biotech, San Diego, CA, United States) analyzed as in ref. (45). Genes were considered significantly expressed when showing a present call in at least 50% of the experiments and a *P*-value < 0.05 (by ANOVA test) between samples. A False Discovery Rate < 10% was used to adjust *P*-values. The resulting gene lists underwent clustering using Cluster 3.0 (TreeView, Stanford University Labs), and IPA was used to identify functions and pathways associated with up-/downregulated genes.

### Phagocytosis

Phagocytosis of *P. aeruginosa* by human and mouse phagocytes was assessed using two different fluorescent strains of *P. aeruginosa.* The PA01 strain modified to stably express GFP (kindly provided by GB. Pier, Harvard Medical School, Boston) was grown to sub-confluence (as described above), washed, and suspended at ∼4 × 10^7^ colony-forming units (CFU)/mL in DPBS. For a separate set of experiments, the RP73 strain conjugated with the acid-sensitive probe pHrodo Red (Thermo Fisher) was used. Briefly, RP73 (60 mg, dry pellet) was suspended at 20 mg/mL in 100 mM NaHCO_3_ and incubated 60 min with 0.5 mM pHrodo at r.t. Cells were spun down (2,700 rcf, 5 min), washed thoroughly three times to remove the excess of dye, and suspended at 1–1.5 mg/mL in DPBS (pH 7.2–7.4).

Lungs of CF mice were gently dissociated with the GentleMACS and cells (1 × 10^5^/200 μL of DPBS) were treated (15 min, 37°C) with vehicle or RvD1 and infected with GFP-PA01 (∼2 × 10^6^ CFU). After 60 min at 37°C on a rotating wheel, cells were spun down (300 rcf, 5 min) and stained with anti-CD45, CD11b, F4/80 antibodies (from Biolegend) prior to acquisition on a flow cytometer that was used to measure the percentage of GFP^+^ MΦ.

Phagocytosis of *P. aeruginosa* by human PMN was investigated incubating 50 μL of peripheral blood (with Na citrate or K_3_EDTA as anti-coagulant) with vehicle or RvD1 (15 min, 37°C) and of pHrodo-RP73 (5 μL, 30 min). Following hypotonic lysis of erythrocytes cells were suspended in DPBS and analyzed on a flow cytometer to determine the percentage of pHrodo^+^ PMN.

To assess phagocytosis by sputum phagocytes, cells were obtained upon dissociation (40 sec) of sputum with the GentleMACS dissociator and suspended in DPBS (at 1 × 10^5^/200 μL). Cells were treated with vehicle or RvD1, infected with pHrodo-RP73 (5 μL) for 30 min at 37°C, after which cells were washed and stained with an anti-CD11b fluorescent antibody. Phagocytosis was determined with flow cytometry measuring the percentage of pHrodo^+^ phagocytes that were identified as PMN or MΦ, based on CD11b expression, size, and granularity.

Phagocytosis of RP73 by human blood monocyte-derived MΦ was carried out as in refs. (3, 36). Cells were kept in GM-CSF-free medium 72 h before experiments as a washout time to remove the effect of this growth factor on phagocytosis.

Each tested condition was assayed as triplicate. In all cases, negative controls were included and consisted in cells that were not treated with labeled bacteria (used to set the cell background fluorescence on the flow cytometer) and cells treated with GFP-bacteria and kept at 4°C throughout the experimental procedures (used to subtract the GFP fluorescence arising from bacteria that were bound but not internalized by leukocytes).

### Statistics

Results were reported as arithmetic mean ± SE, unless otherwise indicated. Statistical analysis of *in vivo* and *in vitro* experiments was carried out using one-way ANOVA followed by Holm–Sidak or Dunn’s *post hoc* test, as appropriate, for the distribution of variance among groups. CFBEC and MΦ experiments and real time PCR data were analyzed with paired t-test. Survival curves and weight loss grade among vehicle- or RvD1-treated infected mice were compared using LogRank Mantel-Cox and one-way ANOVA test respectively. *P*-values < 0.05 were considered statistically significant.

### Study Approval

The study involving volunteers with CF were approved by the local Ethic Committee at the University of Chieti (Prot. 2301/2017) and conducted according to Declaration of Helsinki principles. Written informed consent was received from participants prior to inclusion in the study.

Experimental procedures with mice were approved by the Italian Ministry of Health (Approval #855/2017-PR and 13/2018-PR) in accordance to National laws (D. Lgs 26/2014).

## Results

### RvD1 Reduces *P. aeruginosa*-Induced Lung Infection, Inflammation, and Damage in CF Mice

**FIGURE 1:**
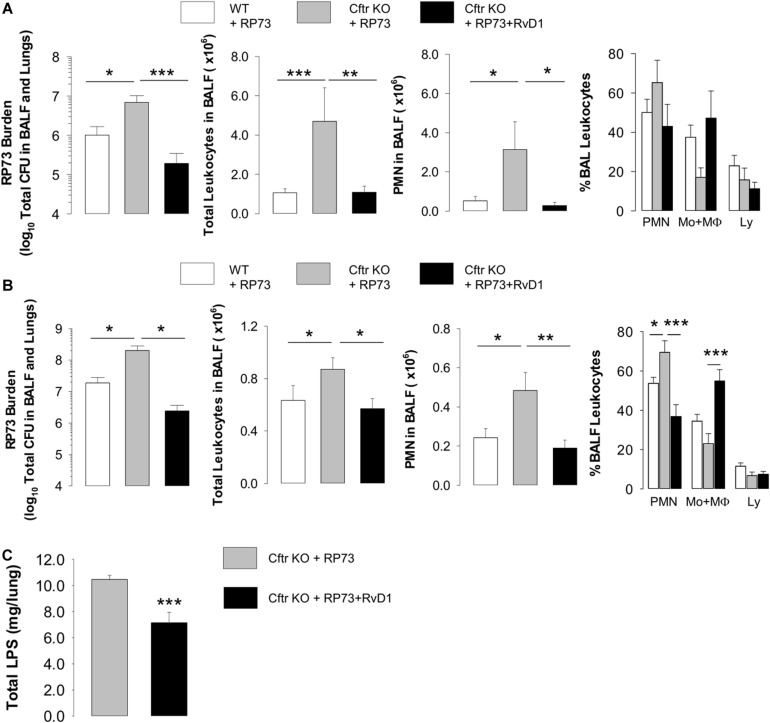
RvD1 reduces *P. aeruginosa*-induced lung inflammation in CF mice. **(A)** Total lung bacterial titer, leukocyte count, PMN number, and percentage of total BAL leukocytes quantified at 5 DPI in *Cftr* KO mice inoculated with agar-embedded RP73 and treated with vehicle (0.05% EtOH) or RvD1 (100 ng), and vehicle-treated WT littermates. Mice received daily treatments from 3 h up to 4 DPI. Results are mean values ± SE (*n* = 6–8 mice/data point and repeated twice with independent experiments). **(B)** *P. aeruginosa* burden, total leukocyte number, PMN count, and percentage of total BAL leukocytes determined in CF or WT mice infected with agar-immobilized RP73 randomized to receive vehicle (0.05% EtOH) or RvD1 (100 ng) three times/week via gavage from 5 to 21 DPI. Results are mean values ± SE (*n* = 8–11 mice/data point from two independent experiments). **(C)** LPS levels measured in cell-free lung homogenates from vehicle- or RvD1-treated CF mice at 21 DPI. Results are mean ± SE from nine mice/data point. *P < 0.05; **P < 0.01; ***P < 0.001 (one-way ANOVA).

Given the essential role of non-resolving inflammation in the pathogenesis of CF lung disease and the actions of RvD1 (3–5), we tested RvD1 in preclinical models that recapitulate human CF lung disease (46) and are widely used and recommended for testing new drugs (47). To this end, we used the RP73 *P. aeruginosa* to induce a chronic lung infection in *Cftr*-KO and WT mice. RP73 is a clinical strain isolated from a patient with CF at a late stage of infection. Compared to other laboratory strains (e.g., PA01), RP73 is more resistant to antibiotics used to treat infections in patients with CF (3) and is endowed with pathogenic traits that permit the establishment of persistent colonization in human and murine lungs (48). As shown in Figure 1A, at 5 DPI, RP73 titer, total leukocyte and PMN counts in BALF were significantly higher in *Cftr* KO mice compared to WT, suggesting a defect in bacterial clearance and hyper-responsiveness to infection of CF mice. Administration of RvD1 (100 ng/day, from 3 h post infection up to 4 DPI) significantly diminished bacterial load, total leukocyte, and PMN number in BALF and gave a downward trend in the percentage of PMN and lymphocyte and a slight increase in monocyte/MΦ in BALF (Figure 1A). To assess whether RvD1 promoted resolution of *P. aeruginosa*-induced chronic infection once established, mice were infected, randomized to vehicle or RvD1 (100 ng, 3 times/week, *per os*) on day 5, and monitored up to 21 DPI. As shown (Figure 1B), RP73 load was > 10^7^CFU in both WT and CF mice, confirming that inclusion into agar beads enables the persistence of *P. aeruginosa* infection in mouse lungs as we demonstrated previously (3). RvD1 dampened bacterial burden down to ∼10^6^ total CFU in CF mice, which had a ∼1 log order higher bacterial titer compared to WT (Figure 1B). CF mice also exhibited a markedly higher inflammatory response to *P. aeruginosa* late chronic infection compared to WT animals, as indicated by a significant increase in BALF total leukocytes as well as PMN accumulation (Figure 1B). Treatment of CF mice with RvD1 reverted this phenotype, reducing infiltrated leukocytes and neutrophils, diminishing the percentage of PMN, and augmenting monocyte/MΦ in BALF (Figure 1B). Moreover, RvD1 significantly reduced LPS concentrations in BALF, which suggests its ability to activate removal of bacterial cells as well as inflammatory endotoxins from infected airways (Figure 1C).

**FIGURE 2:**
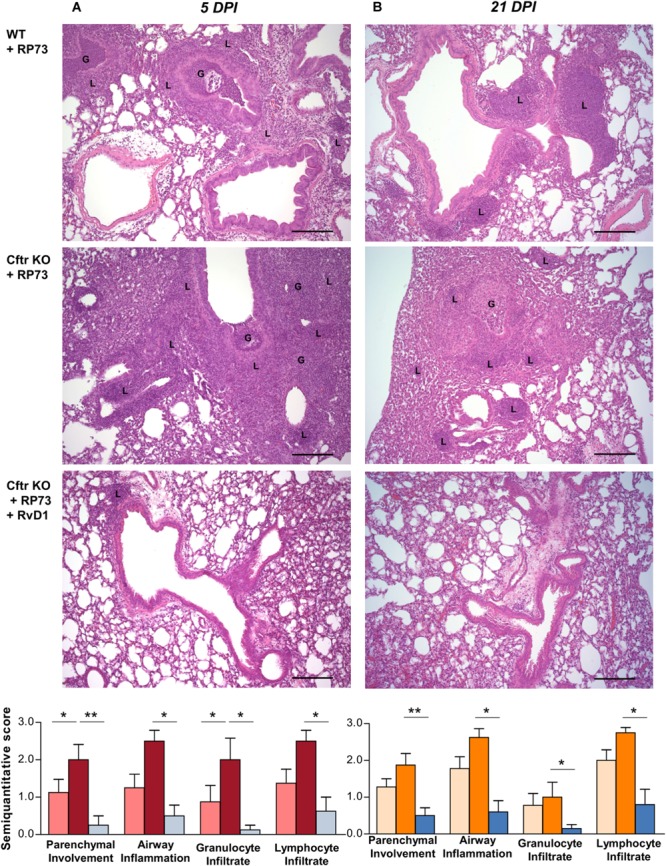
RvD1 improves chronic *P. aeruginosa* lung infection and inflammation in CF mice. **(A)** Hematoxylin/eosin (H/E)-stained microsections and semiquantitative scores for histological parameters of lung inflammation at 5 DPI or 21 DPI. Photomicrographs are representative of 6–8 mice/data point (from two independent experiments). **(B)** Semiquantitative scores are expressed as mean ± *SE* from 8 mice/data point. G, granulocytes; L, lymphocytes; *P < 0.05; **P < 0.01 (one-way ANOVA). Scale bars, 100 μm.

Protective effects of RvD1 were observed at the tissue level in *Cftr* KO mice, in which RvD1 significantly reduced parenchymal damage as well as airway inflammation and granulocyte and lymphocyte infiltration as determined by histological examination of lung microsections at both 5 (Figure 2A) and 21 DPI (Figure 2B).

Collectively, these results demonstrate the efficacy of RvD1 to limit the excessive inflammatory response and lung damage in CF mice undergoing chronic *P. aeruginosa* infection without impairing microbial clearance.

### RvD1 Controls Chemical Mediators in Chronically Infected CF Mice

**FIGURE 3:**
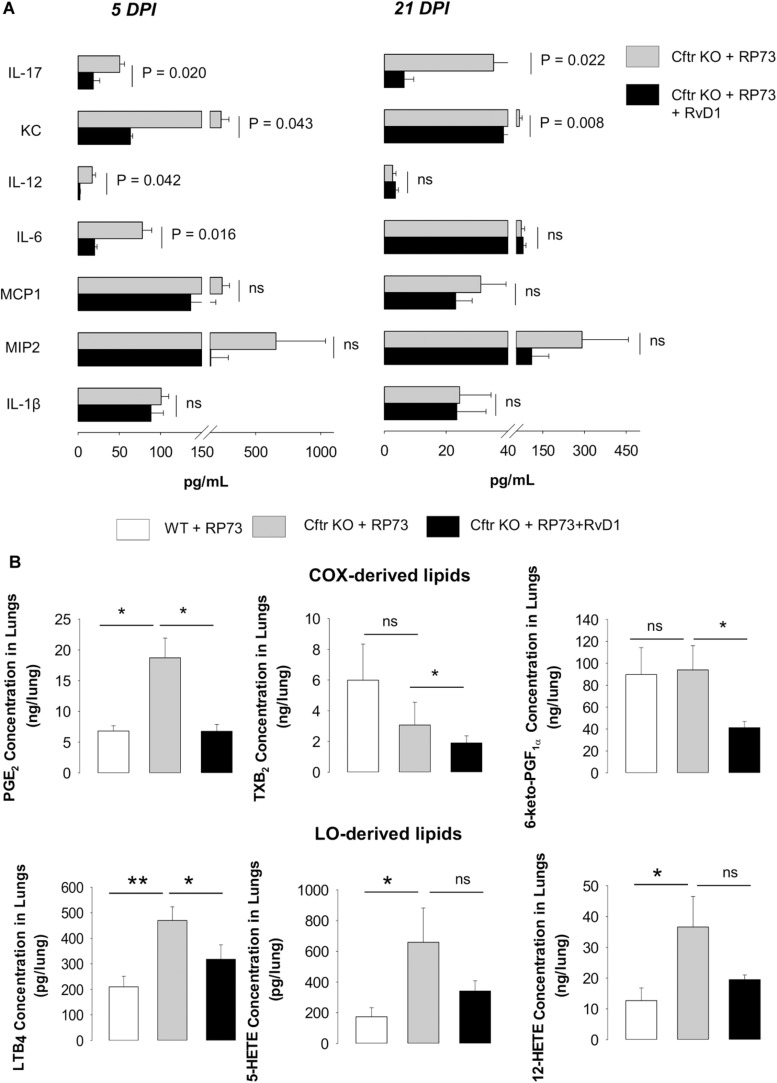
RvD1 reduces inflammatory mediators in CF mice undergoing chronic *P. aeruginosa* infection. **(A)** Levels of cytokines and chemokines determined in lung homogenates collected at 5 or 21 DPI from CF mice infected with agar-embedded RP73 and treated with vehicle or RvD1. Results are mean ± SE from eight mice/group. Significance was determined with one-way ANOVA test. **(B)** Cyclooxygenase (COX)- and lipoxygenase (LO)-derived lipid mediators identified and quantified using by LC-MS/MS in lung homogenates collected from CF mice 21 days following challenge with RP73. Results are expressed as mean ± SE of ng/sample from five mice/group. *P < 0.05. **P < 0.01 (one-way ANOVA). PG, prostaglandin; LT, leukotriene; TX, thromboxane; HETE, hydroxyeicosatetraenoic acid.

Given these findings, we sought to determine whether RvD1 regulates soluble mediators involved in leukocyte recruitment and inflammation. Treatment with RvD1 significantly reduced KC (a major PMN chemoattractant and the murine analog of IL-8) and IL-17 (a cytokine involved in CF lung pathology) in lung homogenates of CF mice, both at 5 and 21 DPI, whereas IL-12 and IL-6 were significantly lower in RvD1-treated animals at 5 DPI (Figure 3A). Among lipid mediators, prostaglandin (PG) E_2_ and LTB_4_, which are markedly elevated in patients with CF and contribute to lung disease (18, 19), were higher in CF mice lungs compared to WT, as were 5- and 12-hydroxyeicosatetraenoic acid (HETE). Of interest, RvD1 administration significantly lowered PGE_2_ and LTB_4_, as well as thromboxane (TX) B_2_ and 6-keto-PGF1_α_ (Figure 3B).

Hence, RvD1 reduces chemical mediators that drive leukocyte infiltration, chronic inflammation, and lung pathology.

### RvD1 Enhances Resolution of Infection and Inflammation in CF Mice

**FIGURE 4:**
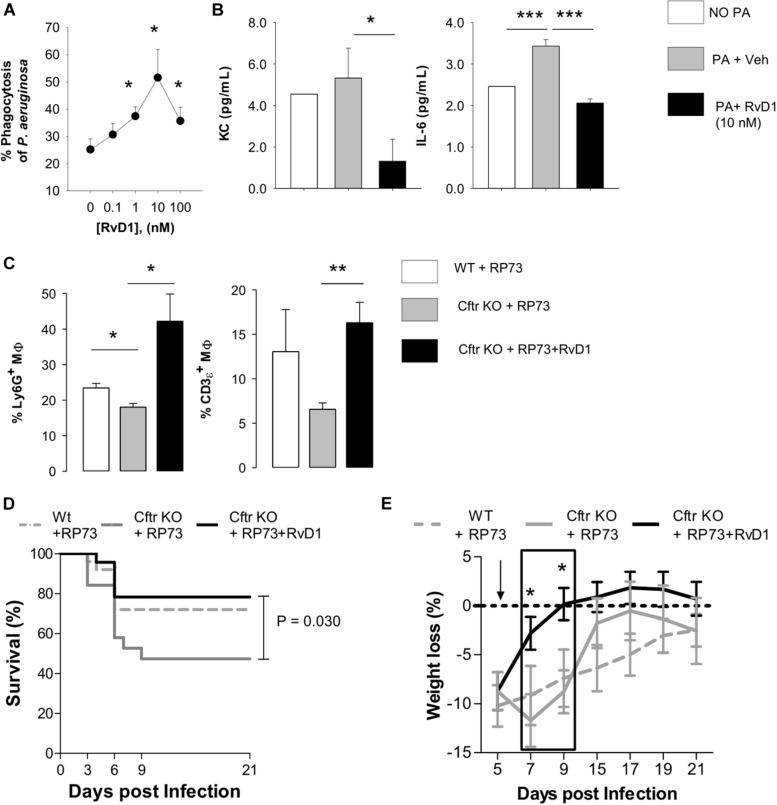
RvD1 enhances clearance of *P. aeruginosa* and efferocytosis by lung MΦ while dampening inflammation and improving health in chronically infected CF mice. **(A)** MΦ (F4/80 + cells) obtained from lungs of CF mice upon aseptic dissociation, treated with vehicle (0.05% EtOH) or RvD1 and infected (60 min, 37°C, 5% CO_2_) with 2 × 10^6^ CFU of GFP-expressing *P. aeruginosa*. MΦ were collected by centrifugation, fixed, and analyzed using flow cytometry to assess phagocytosis by measuring the percentage of GFP^+^ cells. MΦ treated with GFP-*P. aeruginosa* as above and kept at 4°C throughout the experiment were used as a control to determine the number of fluorescent bacteria that were bound but not internalized by leukocytes. Results are mean ± SE from five separate experiments. *P < 0.05 (one-way ANOVA). **(B)** Levels of KC and IL-6 in cell-free supernatants collected from incubations described above and measured using a commercial ELISA. Results are mean ± SE from five separate experiments *P < 0.05; ***P < 0.001 (one-way ANOVA test). **(C)** *In vivo* efferocytosis of MΦ in BAL from RP73 infected mice. MΦ collected from RP73-infected mice 21 DPI were stained with an anti-F4/80 antibody, fixed-permeabilized, and counterstained with Ly6G (a PMN marker) and CD3ε (a T cell marker). Percentages of Ly6G or CD3ε-positive MΦ were determined with flow cytometry. Unpermeabilized MΦ counterstained as above was used as a control of non-specific binding of anti-Ly6G and CD3ε antibodies. Results are mean ± SE from five mice/data points obtained from four separate experiments. *P < 0.05; **P < 0.01 (one-way ANOVA). Differences in survival (**D**) and weight loss (**E**) in RP73-infected mice. Results are mean values ± SE (*n* = 8–11 mice/data points from two independent experiments). Survival curves and weight changes were compared with LogRank-Mantel Cox and one-way ANOVA tests respectively. *P < 0.05 versus *Cftr* KO mice + RP73.

To determine mechanisms underlying the reduction in *P. aeruginosa* colonization and resolution of infection in CF lungs by RvD1, we assessed phagocytosis. Lung MΦ obtained from CF mice were treated with RvD1 or vehicle, incubated with GFP-expressing *P. aeruginosa*, and counterstained to measure the percentage of cells with engulfed bacteria by flow cytometry. RvD1 increased phagocytosis of *P. aeruginosa* in a concentration-dependent manner (Figure 4A). Of interest, RvD1 also significantly decreased the release of KC and IL-6 (Figure 4B), indicating that the stimulation of phagocytic activity did not trigger further inflammatory signaling in MΦ, in line with *in vivo* results.

Furthermore, since reduction in infiltrated leukocytes in RvD1-treated mice could arise from increase in efferocytosis, a paramount MΦ action in resolution, we measured if RvD1 activated this essential pro-resolution process. Flow cytometric analysis of BALF cells indicated that *Cftr* KO MΦ had a lower amount of ingested PMN (Ly6G^+^ cells) and a downward trend in intracellular content of lymphocytes (CD3ε^+^ cells) compared to WT counterparts, indicative of decreased efferocytosis in CF mice. Remarkably, we found a significantly higher percentage of MΦ with ingested PMN and T lymphocytes in RvD1-treated CF mice (Figure 4C). Hence, RvD1 limits leukocyte accumulation during *P. aeruginosa* infection by both reducing chemoattractant and inflammatory mediators and stimulating their removal by MΦ via efferocytosis. Finally, survival of infected CF mice, which was shorter compared to non-CF littermates, and recovery of initial weight after infection were significantly improved by RvD1 (Figures 4D,E), thus corroborating its beneficial pro-resolution activities.

### RvD1 Stimulates Microbial Clearance and Dampens Inflammation in Cells From Volunteers With CF

**FIGURE 5:**
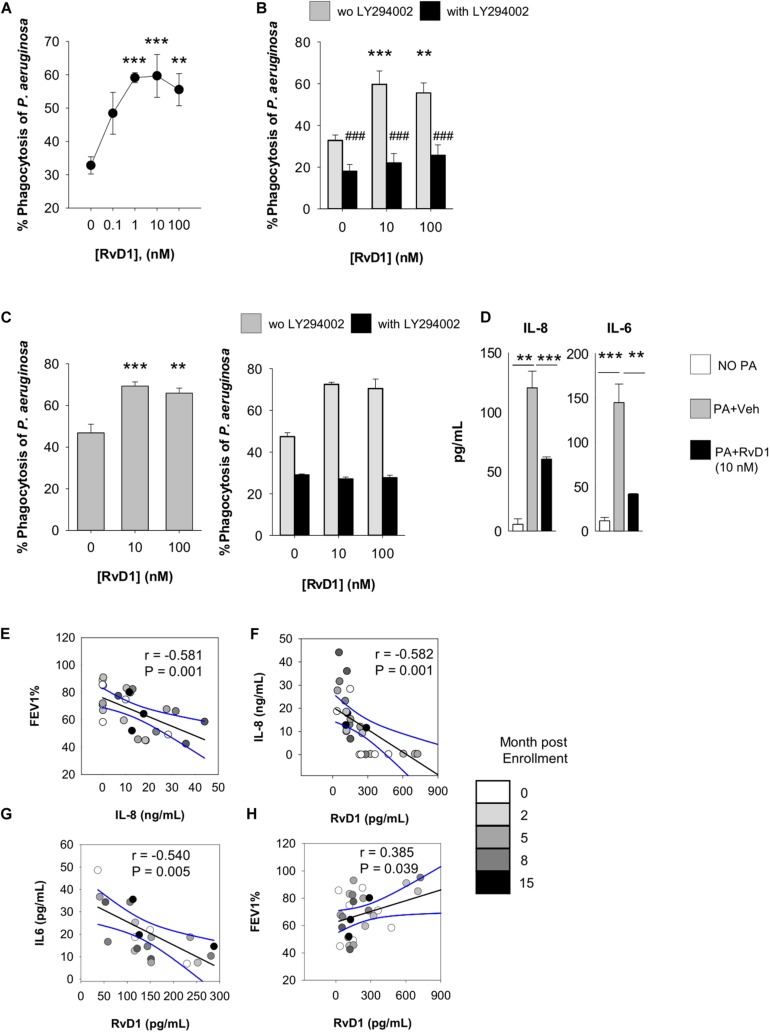
Actions of RvD1 on phagocytes from volunteers with CF. Phagocytosis of *P. aeruginosa* by peripheral blood PMN **(A,B)** and PMN and MΦ in sputum **(C)** of volunteers with CF. Cells were treated (15 min, 37°C) with RvD1 or vehicle (0.05% EtOH) and phrodo-RP73 (30 min, 37°C). Phagocytosis was assessed with flow cytometry by determining the percentage of fluorescent PMN/MΦ that was gated based on size/granularity and CD11b expression. LY294002 (50 μM) was added 15 min prior to RvD1 or vehicle to inhibit PI3K activity. Results are mean ± SE from experiments with cells from six different volunteers. **P < 0.01; ***P < 0.001 versus vehicle; ###*P* < 0.001 versus the corresponding treatment group without LY294002 (one-way ANOVA). The right graph in panel **(C)** shows a representative result from experiments with six different volunteers, with error bars indicating SE of determinations carried out as a triplicate. **(D)** Levels of cytokines/chemokines released by sputum leukocytes from study participants (2 × 10^5^ cells), treated with RvD1 or vehicle (15 min, 37°C) prior infection with *P. aeruginosa* (1 × 10^6^). Results are mean ± SE from experiments with cells from 3 different study participants. **P < 0.01 versus cells incubated with *P. aeruginosa* plus vehicle (one-way ANOVA). **(E–H)**. Correlations between FEV_1_ and bioactive mediators quantified in sputum samples. Black lines represent Pearson’s correlations (with *r* coefficients and *P*-values indicated within each plot). Dashed lines are 95% confidence interval.

For translation to human pathology, we determined if RvD1 enhanced anti-*P. aeruginosa* responses by PMN from volunteers with CF. To this end, we used RP73 conjugated with pHrodo, a fluorogenic dye that increases fluorescence when labeled bacteria are internalized into phagosomes, and the pH becomes acidic due to the release of lysosome content, thus allowing the direct assessment of phagocytic activity and phagolysosome maturation. As shown, nanomolar RvD1 significantly increased phagocytosis of pHrodo-RP73, as determined by the percentage of fluorescent PMN (Figure 5A). Individual responses to RvD1 of PMN from each study participant are reported in Supplementary Figure 1A.

This enhancement was diminished in the presence of LY294002 (Figure 5B), an inhibitor PI3K, which is required for effective phagocytosis, resolution of inflammation, and pro-resolving actions of SPM (49). Moreover, to obtain clues of RvD1 bioactions on leukocytes present in the airways of patients, we used sputum as a suitable source of cellular components of airway inflammation in CF (50). PMN and MΦ in sputum stained positive for the RvD1 receptors ALX/FPR2 and GPR2 (Supplementary Figure 1B), and *ex vivo* RvD1 gave a concentration-dependent increase in phagocytosis of pHrodo-RP73 (Figure 5C) that was also blunted by LY294002 as in blood PMN.

In line with results obtained with mouse cells, RvD1 also gave a significant reduction in IL-8 and IL-6 release by sputum phagocytes during phagocytosis of *P. aeruginosa* (Figure 5D), indicating that RvD1 enhances anti-*P. aeruginosa* defense actions while it dampens PMN and MΦ inflammatory responses that can amplify leukocyte infiltration and have detrimental effects in the airways of patients with CF. Representative histogram plots from flow cytometry results showing shift in fluorescence of pHRodo are reported in Supplementary Figure 1C.

To corroborate these findings, we assessed the correlation between sputum concentrations of RvD1 and these cytokines, known to worsen the progression of the inflammatory process in CF (18, 51). To this end, sputa were obtained from adult, clinically stable, volunteers with CF and without CF (Table 1). Concentrations of RvD1 and IL-6 in sputum were not significantly different between the two groups of participants, whereas IL-8 was significantly more abundant in volunteers with CF and RvD1/IL-8 ratios were significantly lower in this cohort of study participants, in line with previous observations (18, 34) (Table 2). To account for the variability of concentrations of soluble mediators in relation to the clinical status, samples were collected at baseline and after 2, 5, 8, and 15 months. As shown (Figure 5E), FEV_1_ decreased linearly with the increment in IL-8 sputum concentrations, consistently with the role of this chemokine in the progression of CF lung disease (18). In addition, RvD1 sputum concentrations were inversely correlated with those of IL-8 (*P* = 0.001) and IL-6 (P = 0.004) and directly with FEV_1_ (*P* = 0.038) (Figures 5F–H). Together, these results suggest that RvD1 enhances microbial clearance by leukocytes in pulmonary secretions and dampens inflammatory responses and mediators that promote lung disease in patients with CF.

**TABLE 1:** Demographic and clinical characteristics of study participants.

**Age/sex**	**CFTR genotype**	**Ethnicity**	**FEV_1_%**	**Modulator therapy**	***P. aeruginosa***
38/M	ΔF508/ΔF508	White	80	Orkambi	Positive
18/M	ΔF508/ΔF508	White	55	Orkambi	Positive
32/F	ΔF508/ΔF508	White	50	Orkambi	Positive
23/M	ΔF508/ΔF508	White	86	Orkambi	Positive
43/M	G85E/R75X	White	60	None	Positive
28/M	ΔF508/R553X	White	54	None	Positive
35/F	ΔF508/N1303K	White	58	None	Positive
28/F	G542X/2183AAG	White	89	None	Positive
18/M	ΔF508/ΔF508	White	71	Orkambi	Positive
23/F	ΔF508/ΔF508	White	85	Orkambi	Positive
21/F	ΔF508/W1282X	White	54	None	Positive
34/M	Wild type	White	–	–	–
38/M	Wild type	White	–	–	–
41/M	Wild type	White	–	–	–
30/F	Wild type	White	–	–	–
45/M	Wild type	White	–	–	–
35/F	Wild type	White	–	–	–
34/F	Wild type	White	–	–	–
39/F	Wild type	White	–	–	–

*FEV_1_ values are reported as the mean values in the 6 months prior to enrollment.*

**TABLE 2:** Sputum concentrations of RvD1, IL-8, and IL-6 in study participants with and without CF.

	**CF**		**Non-CF**		***P*-value**
	**Mean**	**SE**	**Mean**	**SE**	
RvD1 (pg/mL)	199.00	63.62	226.08	35.87	0.785
IL-8 (ng/mL)	822.20	431.13	6.54	4.84	**0.005**
IL-6 (pg/mL)	68.44	16.79	53.67	30.69	0.216
RvD1/IL-8 (×10^–3^)	12.02	5.27	144.92	29.29	**0.004**

*The significant *P*-values are indicated in boldface.*

### RvD1 Regulates the Inflammatory Transcriptome and Signaling in CF MΦ and Epithelial Cells

Since MΦ and bronchial epithelial cells play essential roles in the pathophysiology of CF (20), we sought to determine whether RvD1 modifies genes involved in inflammation and response to infections. To this end, a genome-wide microarray-based analysis was carried out with human peripheral blood monocyte-derived CF MΦ and fully differentiated CFBEC subjected to *P. aeruginosa* infection. Notably, RvD1 enhanced phagocytosis of *P. aeruginosa* by monocyte-derived MΦ and reduced CFU on apical surfaces of CFBEC (Supplementary Figure 2B), confirming *in vivo* results and providing basis for analysis of gene expression.

**FIGURE 6:**
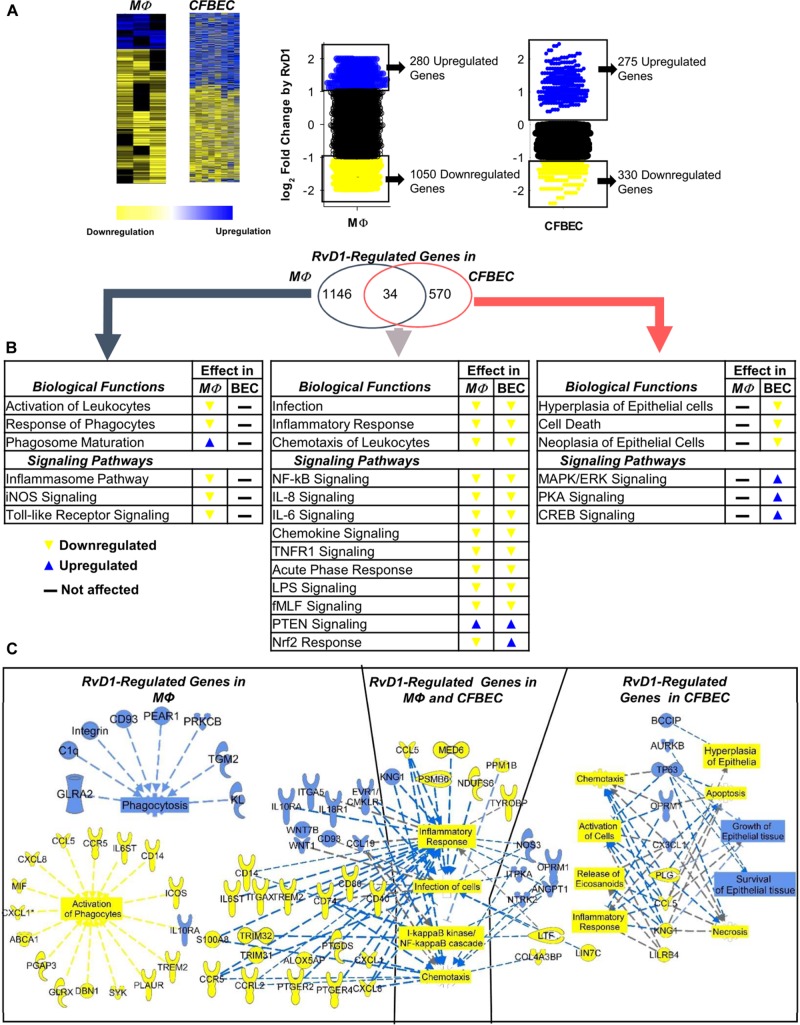
RvD1 regulates select genes related to microbial clearance and inflammatory signaling in CF MΦ and epithelial cells. **(A)** Homozygous ΔF508 peripheral blood monocyte-derived MΦ (0.5–1 × 10^6^ cells/plate) and CFBEC (2.5 × 10^6^ cells/plate) were treated with RvD1 (10 nM) or vehicle (0.01% EtOH) for 15 min at 37°C and infected with RP73 *P. aeruginosa* (∼7.5 × 10^6^ CFU/plate). Medium was removed after 3 h, and total RNA was used for microarray analysis. Shown here are heat map view (Cluster 3.0, TreeView, Stanford University Labs) and scatter plots of gene expression patterns (mean from five different donors). **(B)** IPA findings of biological functions associated with RvD1-regulated genes in MΦ and CFBEC. **(C)** Top RvD1-regulated genes and associated functions identified by IPA. Blue symbols, upregulated genes; yellow symbols, downregulated genes; blue dotted lines, expression leading to activation; yellow dotted lines, expression leading to inhibition; gray dotted lines, expression leading to unpredictable effect of function; blue boxes, activated functions; yellow boxes, inhibited functions.

As shown in Figure 6A, RvD1 strikingly modified the whole transcriptome of CF cells. Ingenuity Pathway Analysis (IPA) revealed that genes downregulated by RvD1 in both MΦ and CFBEC were significantly associated to infection, inflammatory response, and leukocyte chemotaxis. Functions and pathways uniquely regulated in MΦ encompassed activation of leukocytes, maturation of phagosome, and inflammasome response. In CFBEC, RvD1-modified functions were mostly related to cell growth or death, while signaling pathways of several kinases and CREB were activated (Figure 6B and Table 3).

**TABLE 3:** List of RvD1 regulated genes.

**Symbol**	**Entrez gene name**	**Fold change**	**Type(s)**
ALOX5AP	Arachidonate 5-lipoxygenase activating protein	–1.284	Other
AURKB	aurora kinase B	1.811	Kinase
BCCIP	BRCA2 and CDKN1A interacting protein	1.701	Other
CCL19	C-C motif chemokine ligand 19	1.427	Cytokine
CCL5	C-C motif chemokine ligand 5	–1.410	Cytokine
CCR5	C-C motif chemokine receptor 5	–1.996	G-protein coupled receptor
CCRL2	C-C motif chemokine receptor like 2	–1.558	G-protein coupled receptor
CD14	CD14 molecule	–1.453	Transmembrane receptor
CD40	CD40 molecule	–1.091	Transmembrane receptor
CD74	CD74 molecule	–1.293	Transmembrane receptor
CD80	CD80 molecule	–1.776	Transmembrane receptor
CD93	CD93 molecule	1.742	Other
CMKLR1	chemerin chemokine-like receptor 1	1.985	G-protein coupled receptor
CX3CL1	C-X3-C motif chemokine ligand 1	0.701	Cytokine
CXCL1	C-X-C motif chemokine ligand 1	–1.496	Cytokine
CXCL8	C-X-C motif chemokine ligand 8	–1.343	Cytokine
IL10RA	interleukin 10 receptor subunit alpha	1.73	Transmembrane receptor
IL6ST	interleukin 6 signal transducer	–1.336	Transmembrane receptor
ITGA5	integrin subunit alpha 5	1.479	Transmembrane receptor
MIF	macrophage migration inhibitory factor	–1.626	Cytokine
NOS3	nitric oxide synthase 3	1.313	Enzyme
OPRM1	opioid receptor mu 1	0.776	G-protein coupled receptor
PTGDS	prostaglandin D2 synthase	–1.674	Enzyme
PTGER2	prostaglandin E receptor 2	–1.473	G-protein coupled receptor
PTGER4	prostaglandin E receptor 4	–1.702	G-protein coupled receptor
S100A8	S100 calcium binding protein A8	–1.179	Other
TGM2	transglutaminase 2	1.043	Enzyme
TP63	tumor protein p63	1.811	Transcription regulator
WNT1	Wnt family member 1	1.925	Cytokine

*See Supplementary Table 2 for the full list of genes.*

In MΦ, RvD1 downregulated genes related to inflammation, like chemokines (RANTES/CCL5, IL-8/CXCL8, and CXCL1), surface molecules (CD14, CD40, CD80, and CCR5), PGE_2_ receptors [(PTEGR) 2 and 4], and the 5-LO-activating protein (ALOX5AP), which drives LTB_4_ synthesis and MΦ activation. On the contrary, CD93, IL10 receptor α (IL10RA), CD93, and the Wnt family member 1 and 7B (WNT1/7B), which enhance phagocytosis and reduce the inflammatory response, were upregulated (Figure 6C and Table 3).

We identified CCL5 as one of the genes linked to inflammation, NF-κB, and leukocyte chemotaxis, which were downregulated by RvD1 in CFBEC. In contrast, the tumor protein 63 (TP63), opioid receptor μ 1 (OPRM1), and aurora kinase B (AURKB), known to promote cell survival, dampen cell hyperplasia, and regulate epithelial cell growth, were significantly upregulated (Figure 6C).

**FIGURE 7:**
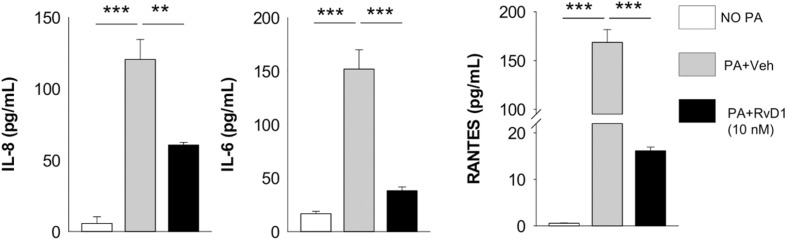
RvD1 dampens inflammatory cytokine release by MΦ from volunteers with CF. Levels of chemokines and cytokines were measured, using a commercial ELISA kit, in cell-free supernatants collected from RvD1-treated CF peripheral blood monocyte-derived MΦ during *P. aeruginosa* infection. **P < 0.01; ***P < 0.001 (one-way ANOVA). PA, *P. aeruginosa*.

In keeping with these results, RvD1 treatment significantly reduced protein levels of IL-8, RANTES, and IL-6 in human CF MΦ infected by *P. aeruginosa*, providing functional readouts of transcriptomic analyses (Figure 7).

Therefore, RvD1 downregulates the inflammatory transcriptome and signaling in CF cells during *P. aeruginosa* infection that can sustain the exaggerated inflammation in CF lungs.

## Discussion

CF is an autosomal recessive genetic disease characterized by early, exaggerated, non-resolving inflammation as well as recurrent airway infection, resulting in progressive lung disease and death. Although improvements in antibiotics and pulmonary therapies have considerably contributed to increase the median life expectancy to ∼40 years, and CFTR modulators have demonstrated some efficacy in individuals carrying selected mutations, there is a paramount need of new therapeutics that can promote resolution of inflammation, clearance of infection, and lung tissue repair (13). Here, we report that administration of RvD1 to CF mice undergoing chronic *P. aeruginosa* lung infection exerts protective effects *in vivo*, limiting neutrophil infiltration, lung inflammation, and bacterial load, while enhancing phagocytosis and reducing organ damage. In human cells from volunteers with CF, RvD1 enhanced phagocytosis of *P. aeruginosa*, reduced the release of inflammatory mediators by sputum leukocytes, and dampened inflammatory genes in both macrophages and bronchial epithelial cells. RvD1 carries multipronged beneficial effects on lung inflammatory diseases. Work from Eickmeier and colleagues was one of the first showing beneficial roles in acute lung injury (7), while our previous work was the first demonstrating enhancement of resolution of chronic lung infection by RvD1 (3). Together, results presented here extend previous findings showing protective actions of RvD1 in the lung system (3, 4) and give foundation for RvD1 as a prototypic pro-resolutive therapeutic strategy to reduce the burden of non-resolving inflammation and infections in CF.

Preclinical testing of new anti-inflammatories for CF should be carried out with animals that most closely mimic the clinical phenotype. While ferrets and larger animals are significantly better at reflecting human pathology, their use is resource-intensive, time-consuming, and not suitable for pharmacological tests. The mouse colony used in this study, along with the induction of pulmonary infection with bacteria embedded into agar beads, is a suitable model for preclinical studies, since it enables *in vivo* studies with persistent lung infection and inflammation displaying cellular, biochemical, and histological hallmarks observed in patients with CF, including a heightened inflammatory response similar to that observed in patients. The use of a clinical *P. aeruginosa* strain, endowed with adapted virulence, ability to establish persistent infections and to resist to several classes of antibiotics, also increases the clinical relevance of the experimental approach used in our study. In CF mice, we found a significantly higher bacterial load, PMN accumulation in BALF, and lung damage, compared to WT littermates (Figures 1, 2), which further corroborates the suitability of this mouse strain as a preclinical model for CF drug discovery. We also observed that nanogram doses of RvD1 induced a significant phenotype shift in infected CF animals, reducing the number of BALF neutrophils, lung inflammation, and organ histopathology scores to reach levels observed in WT mice (Figures 1, 2). In this experimental setting, RvD1 reduced pro-inflammatory and chemotactic proteins and lipid autacoids *in vivo*, including LTB_4_, PGE_2_, TXB_2_, KC, IL-6, and IL-17 (Figure 3), which play pivotal roles in leukocyte infiltration and activation. LTB_4_ and IL-8 are potent PMN chemoattractants and are present in higher amounts in lung secretions of patients with CF compared to healthy subject or patients with other respiratory diseases (18, 19, 31). RvD1 directly stops IL-8-driven PMN chemotaxis (9) and counters LTB_4_-induced actin remodeling and integrin activation (11), which are critical steps in PMN migration. Hence, RvD1 can act at multiple levels on PMN chemotaxis to regulate their infiltration in CF airways. IL-6 and IL-17 are secreted by airway cells activated in response to bacterial infection and are present in large concentrations in CF airways (18, 52). Since RvD1 administration resulted in a marked reduction in airway *P. aeruginosa* and LPS load (Figure 1C), it is likely that the decreased concentrations of these cytokines reflect a better control of infection. In CF mice, RvD1 also normalized levels of prostaglandins and thromboxane (Figure 3B), which are key mediators of pain, swelling, leukocyte and endothelial cell activation, and airway remodeling in CF (13). Therefore, RvD1 has multipronged actions on lipid and protein mediators that perpetuate lung inflammation in CF.

In order to occur efficiently and beneficially to the host, resolution of inflammation must include the timely removal of pathogens and excessive infiltrated leukocytes through MΦ phagocytosis. Here, RvD1 enhanced non-phlogistic phagocytosis of *P. aeruginosa* by CF mouse lung MΦ, chronically infected sputum phagocytes from volunteers with chronically infected by *P. aeruginosa* (Figures 4, 5), and MΦ (Supplementary Figure 2A). Results from *in vitro* infections of CFBEC also indicate that RvD1 reduces viable *P. aeruginosa* cells on epithelial surface, suggesting a direct effect on bacterial growth and biofilm formation or an enhancement of bacterial clearance. In this regard, RvD1 did not inhibit bacterial growth in standard *in vitro* test carried out in agar plates (53), whereas Ringholz et al. reported that RvD1 restores airway surface liquid height in human CF bronchial cells, which can lead to increased mucociliary clearance (37). Mechanisms underlying the observed reduction in *P. aeruginosa* burden *in vitro* in CFBEC merit further investigations.

*In vivo*, RvD1 restored efferocytosis by BALF MΦ in *Cftr* KO mice, which was significantly impaired compared to non-CF animals (Figure 4). Thus, RvD1 also activates cellular resolution programs that are inefficient in CF cells. Remarkably, the combined anti-inflammatory, pro-resolving and antimicrobial activities of RvD1 significantly improved the clinical outcome of chronic *P. aeruginosa* lung infection in CF mice, extending survival and reducing disease burden (Figures 4D,E), further supporting a RvD1-based pharmacology to treat CF.

The identification of easily accessible biomarkers of pulmonary inflammation is highly needed in CF. Since bronchoalveolar lavage is an invasive technique, sputum may provide a useful alternative to obtain indications on the status of lung inflammation/infection in adult subjects. A previous study identified RvD1 as surrogate biomarker of respiratory function in CF patients (34). Consistent with this, we found that sputum levels of RvD1 were inversely correlated to IL-6 and IL-8 (Figure 5), which are used as markers of efficacy of anti-inflammatory or anti-microbial treatments for CF (51, 54). Mechanistically, we found that RvD1 stimulated *P. aeruginosa* clearance and dampened IL-6 and IL-8 release by sputum leukocytes (Figure 5), providing the first evidence for direct anti-inflammatory and pro-resolutive RvD1 actions on immune cells present in CF airway secretions. Thus, measurements of IL-6 and IL-8 may represent useful surrogate biomarkers for monitoring RvD1 efficacy in clinical CF studies. RvD1 concentrations used in this study (0.1–100 nM) corresponded to mean values measured in sputum (∼200 pg/mL; range 480–25 pg/mL) that equal to ∼0.5–1.3 nM. We did not observe toxicity (e.g., death, degranulation) in cell-based experiments reported here.

The inflammatory response in lungs of patients with CF is dependent upon aberrant NF-κB activation, which results in an overshooting and prolonged secretion of cytokines, for instance by MΦ, following bacterial stimuli (20–22). Here, we found that RvD1 regulated transcriptomics and signaling in MΦ and epithelial cells of patients with CF exposed to *P. aeruginosa*, reducing genes and proteins related to the NF-κB signaling pathway that drive leukocyte chemotaxis, activation, and cytokine release (Figures 6, 7). These include CD14, CD40, and CD80, which are surface receptors that trigger NF-κB activation, and IL-8, IL-6, and RANTES that are NF-κB-responsive cytokines with major roles in the pathogenesis of CF lung disease. Of interest, some of these NF-κB-related molecules (IL-8, IL-6, and RANTES) were significantly diminished by RvD1 treatment also *in vivo* (Figure 3). In previous studies, we found that RvD1 controls expression of miRNAs (miR-21, −146b, and −155), epigenetic factors (CARM1) and genes (MyD88, TLRs, and cytokines) that regulate both up- and downstream NF-κB signaling (3, 10, 12). Moreover, RvD1 blocks NF-κB nuclear translocation in human cells (10, 11) and blunts the release of IL-8 by CF epithelia (37). Therefore, the counteraction of the NF-κB pathway and downstream genes emerges as the main molecular mechanism by which RvD1 stops excessive inflammation.

In CFBEC RvD1 also modulated the expression of genes (e.g., TP63 and AURKB) related to cell growth that are promoted by inflammation. Whether RvD1 carries protective actions in proliferative lung disorders, like cancer, remains of interest. Collectively, genes and pathways identified herein represent the first molecular fingerprint of RvD1 actions on CF MΦ and epithelia and indicate that this SPM counters the excessive inflammatory signaling in CF.

In summary, here we demonstrate that RvD1 reduces chronic *P. aeruginosa* lung infection, inflammation, and damage promoting resolution *in vivo* in CF mice and enhances microbial clearance while dampening inflammatory responses in human cells. Since emerging evidence indicates that failure to initiate resolution can contribute to the pathogenesis of CF, results shown here can provide the foundation for the development of innovative therapeutics aimed at reducing inflammation-based lung disease in patients with CF by harnessing endogenous pro-resolving mediators and mechanisms.

## Data Availability Statement

The raw data supporting the conclusions of this article will be made available by the authors, without undue reservation, to any qualified researcher.

## Ethics Statement

The studies involving human participants were reviewed and approved by the Comitato Etico dell’Università G. d’Annunzio. The patients/participants provided their written informed consent to participate in this study. The animal study was reviewed and approved by Ministero della Salute.

## Author Contributions

AR conceived overall research, designed and carried out the experiments, and wrote the full manuscript. AR, EI, and DM designed and carried out the experiments and wrote the full manuscript. MC, VM, SP, and EC carried out the cellular and mice infection experiments and data analysis. MD performed the lipidomic analysis. AL, AN, and MI conducted the histopathological analysis. MD’A, SF, and VG carried out the microarray analysis. SP, MD, and PM conducted the recruitment of volunteers, collected, and analyzed samples. MR contributed critical reading of the final version of the manuscript.

## Conflict of Interest

A patent application (Application Number 102020000008251 to AR and MR) containing an invention pertaining to this publication has been filed. The remaining authors declare that the research was conducted in the absence of any commercial or financial relationships that could be construed as a potential conflict of interest.
