# Supplementary material for: Resolvin D1 Reduces Lung Infection and Inflammation Activating Resolution in Cystic Fibrosis
Source: Front Immunol. 2020 Apr 28;11:581. doi: 10.3389/fimmu.2020.00581 (PMC7247852; doi:10.3389/fimmu.2020.00581)
Supplement: Supplementary file 1 [file Data_Sheet_1.docx]

**Resolvin D1 promotes resolution of inflammation in cystic fibrosis**

Elisa Isopi, Domenico Mattoscio, Marilina Codagnone, Veronica Cecilia Mari, Alessia Lamolinara, Sara Patruno, Marco D’Aurora, Eleonora Cianci, Annalisa Nespoli, Sara Franchi, Valentina Gatta, Marc Dubourdeau, Paolo Moretti, Maria Di Sabatino, Manuela Iezzi, Mario Romano, and Antonio Recchiuti.

Online data supplement

**Supplementary Materials and Methods.**

#### **P. aeruginosa growth and infections**

The clinical strain of *P. aeruginosa* RP73 (kindly provided by B. Tümmler, Medizinische Hochschule Hannover, Germany), and the genetically modified PA01 strain stably expressing the green fluorescent protein (GFP) (a generous gift of GB Pier, Harvard Medical School, Boston) were grown in tryptic soy broth (TSB) or agar (TSA).

For *in vivo* chronic infection, RP73 was grown in tryptic TSB to mild-log phase (OD_600 nm_ = 0.45 ± 0.05; ~ 2 x10^8^ CFU/mL) and 16 OD (~ 50 ml) were embedded into 100-200 µm diameter tryptic soy agar (TSA) beads that were inoculated intra-tracheally (i.t.) within 24-48 h according to published procedures [1]. Male and female *Cftr* KO [B6.129P2-Cftr^tm1UNC^TgN(FABPCFTR)] mice and WT littermates [2] were obtained from the Cystic Fibrosis animal Core Facility (CFaCore), husbanded in semi-barrier cages, and fed *ad libitum* tap water and chow pellet diet (25/18 CR, Mucedola s.r.l. Settimo Milanese, Italy). Diet contained ~ 4 % fats as a mixture of palmitic (C16:0, 5.0 g/kg), stearic (18:0, 0.8 g/kg), palmitoleic (ω-7 16:1, 0.3 g/kg), oleic (ω-9 18:1, 4.7 g/kg), linoleic (ω-6 18:2, 11.7 g/kg), and linolenic acid (ω-6 18:3, 1.2 g/kg). Mice (8-12 weeks) were infected i.t. with agar-embedded RP73 (~ 3.5 x10^6^ CFU/mouse) for short- and long-term period (5 and 21 days respectively). RvD1 (100 ng/mouse) or equal amount of vehicle (0.5% _vol/vol_ EtOH) were administered via intragastric gavage of 0.2 mL of saline starting at 1 DPI (then daily) or at 5 DPI (then 3 times/week). Mice were monitored daily for clinical signs of disease and those that lost ≥ 20% body weight or showed evidence of severe clinical disease were euthanized before the termination of the experiment.

**BALF and lung analyses**

BALF was collected from mice by injecting 3 aliquots of sterile DPBS i.t. (1 mL each) aspirated with a 22G (0.9 x 25 mm) catheter connected to a 1 mL syringe. Total leukocytes present in BALF were counted using Turk’s solution and stained (15 min, 4°C) with 0.2 µg/5 x 10^5^ cells of fluorochrome-tagged antibodies (all from Biolegend, San Diego, CA) against the following antigens: CD16/32 (clone 93), Ter-119 CD45 (Clone 30-F11), CD11b (clone M1/7), Ly6C (clone HK1.4), F4/80 (clone BM8), Ly6G (clone 1A8), CD3ε (clone 145-2c-11). Samples were analyzed with a FACS Canto II flow cytometer (Becton Dickinson, Milan) and the FACS Diva or FCS Express 6 (DeNovo Software, Glendale, CA) softwares.

Viable RP73 cells in BALF and aseptically dissociated lungs were determined upon serial dilutions (10^-1^ down to 10^-6^), plating on TSA, and overnight growth at 37°C. Cytokines and chemokines were measured with Luminex (Millipore, Vimodrone, Italy) multiplex arrays.

For liquid chromatography-tandem mass spectrometry (LC-MS/MS)-based lipidomics, lungs were rapidly dissociated in ice and snap frozen (at -80 °C) to prevent further degradation of lipid mediators. The extraction protocol and analysis of bioactive lipids were performed as described in Le Faouder, Baillif *et al* [3] and adapted by the Ambiotis SAS (Toulouse, France) standard operating procedures. Samples were taken to solid phase extraction in the presence of deuterated internal standards and lipid mediators (LM) eluted in HCOOMe. After solvent evaporation, samples were dissolved in MeOH and injected into an Agilent 1290 Infinity high performance liquid chromatography (HPLC) system equipped with a Kinetex Biphenyl column (2.1 mm, 50 mm, 1.8 µm) (Phenomenex). LM were eluted with a binary gradient of water/formic acid 0.1 % and acetonitrile/formic acid 0.1 % and taken to MS/MS analysis on a triple quadrupole Agilent 6490 instrument. LM were identified based on matching of retention time to authentic standards. Calibration curves were obtained using authentic LM mixtures and quantification was carried out based on peak areas from multiple reaction monitoring (MRM) transitions.

For histopathology, mouse lungs excised *en bloc* were inflated with 1ml of DPBS to permit even organ expansion (critical for quantitative morphometry), fixed and cut transversally to the trachea into 5.2 mm thick, parallel slabs/lung, starting from the top 2 mm of the lung to ensure uniform random sampling. Slabs were embedded and cut surface-down into 2 mm sections that were stained with H/E (BioOptica, Milan) to detect inflammatory cell infiltrates. Semi-quantitative scores of lung pathology were assigned on a 0–3 scale, based on criteria described in Supplementary Table 1 and in ref. [1].

#### **Phagocytosis**

PA01-GFP was grown to sub-confluence as described above, washed, and suspended at ~4 x10^7^ CFU/mL in DPBS. Lungs of CF mice were gently dissociated with the GentleMACS and cells (1 x10^5^/200 µL of DPBS) were treated (15 min, 37 °C) with vehicle or RvD1 and infected with GFP-PA01 (~2 x10^6^ CFU). After 90 min at 37 °C on a rotating wheel, cells were spun down (300 rcf, 5 min), fixed with 3 % formalin, and stained with anti-CD45, CD11b, F4/80 antibodies (from Biolegend) prior to acquisition on a flow cytometer.

To assess phagocytosis by sputum phagocytes, cells obtained upon dissociation of sputum (40 sec) were suspended in DPBS (1 x10^5^/200 µL), treated with vehicle or RvD1, infected with GFP-PA01 as above and stained with CD45, CD14, and CD11b for flow cytometry analysis. Phagocytosis of GFP-PA01 was determined by measuring the percentage of GFP^+^ MΦ. For assessing phagocytosis by human blood monocyte-derived MΦ, 10-15 OD of RP73 were labeled with the BacLight fluorescent dye (Thermo Fisher Scientific) according to manufacturer’s instruction, washed, and suspended in 1 mL of DPBS. Ten microliters of fluorescent RP73 cells were added to MΦ (1-2 x10^5^/well) seeded in a 24-well plate and treated with vehicle or RvD1 as above. After 90 min at 37 °C, plates were washed twice and the percentage of ingested bacteria was determined by measuring MΦ-associated fluorescence using a plate reader (Synergy H3, Biotek).

#### **Primary CF cell culture, infection, and gene microarray**

CFBEC were isolated from bronchi of patients with the ΔF508/ ΔF508 genotype and provided by Dr. L. Galietta and collaborators (Istituto G. Gaslini, Genoa) as part of the Primary Culture Service of the Fondazione Ricerca Fibrosi Cistica (Verona, Italy). CFBEC were grown on rat tail collagen-coated cell culture Petri dishes in serum free medium (LHC9:RPMI 1640 1:1) with growth factors . For further differentiation into tight epithelia, CFBEC (2.5 x10^6^ cells) were grown in air liquid interface (ALI) conditions on 24 mm Transwell filters (0.4 µm pore Ø) for 8-10 days in differentiation medium (Ham’s F12, 2 % Ultroser G) (Pall Corp., New York) as in [4]

MΦ were differentiated from peripheral blood monocytes isolated as in ref. [5] from de-identified study participants and seeded at 0.5 – 1 x10^6^/plate in 6 well plates 24 to 72 h prior to experiments.

Before infection, CFBEC and MΦ were treated with RvD1 (10 nM) or vehicle for 15 min at 37°C. The infection was established by adding RP73 (~ 7.5 x10^6^ CFU/plate), grown as above, to cells in antibiotic free medium. Medium was removed after 3 h, cells were washed twice, and RNA isolated using the Macherey-Nagel (Düren, Germany) kit. Total RNA extracted from CFBEC and MΦ cells was linearly amplified, labeled with Cy3/5, and hybridized on HOA_007 Human Whole Genome OneArray™ Microarray V7 (29,264 probes, Phalanx Biotech, San Diego) analyzed as in ref. [6].

Genes were considered significantly expressed when showing a present call in at least 50% of the experiments and a p value < 0.05 (by ANOVA test) between samples. A False Discovery Rate < 10 % was used to adjust p values. The resulting gene lists underwent clustering using Cluster 3.0, (TreeView, Stanford University Labs) and IPA to identify functions and pathways associated with up-/downregulated genes. Numbers of viable CFU on the apical surfaces of CFBEC were counted upon lysis of epithelial monolayers infected (3 h, 37 °C) with *P. aeruginosa* using 0.1 % Triton X100/DPBS. Lysates were diluted and plated onto TSA plates.

**Supplementary references**

1 Codagnone M, Cianci E, Lamolinara A, *et al.* Resolvin D1 enhances the resolution of lung inflammation caused by long-term Pseudomonas aeruginosa infection. *Mucosal Immunol* 2018;**11**:35–49. doi:10.1038/mi.2017.36

2 van Heeckeren AM, Schluchter MD, Drumm ML, *et al.* Role of Cftr genotype in the response to chronic Pseudomonas aeruginosa lung infection in mice. *Am J Physiol Lung Cell Mol Physiol* 2004;**287**:L944-52. doi:10.1152/ajplung.00387.200300387.2003 [pii]

3 Le Faouder P, Baillif V, Spreadbury I, *et al.* LC-MS/MS method for rapid and concomitant quantification of pro-inflammatory and pro-resolving polyunsaturated fatty acid metabolites. *J Chromatogr B Anal Technol Biomed Life Sci* 2013;**932**:123–33. doi:10.1016/j.jchromb.2013.06.014

4 Scudieri P, Caci E, Bruno S, *et al.* Association of TMEM16A chloride channel overexpression with airway goblet cell metaplasia. *J Physiol* 2012;**590**:6141–55. doi:10.1113/jphysiol.2012.240838

5 Krishnamoorthy S, Recchiuti A, Chiang N, *et al.* Resolvin D1 binds human phagocytes with evidence for proresolving receptors. *Proc Natl Acad Sci U S A* 2010;**107**:1660–5. doi:10.1073/pnas.0907342107

6 D’Aurora M, Ferlin A, Garolla A, *et al.* Testis Transcriptome Modulation in Klinefelter Patients with Hypospermatogenesis. *Sci Rep* 2017;**7**:45729.

**Supplementary figure Legends**

**Supplementary figure 1**

**A) Individual responses to RvD1 from blood and sputum leukocytes from each study participant.** Phagocytosis of pHRodo-RP73 by blood PMN (*upper plot*) or sputum (*lower plot*) leukocytes in response to RvD1 are shown. Bars indicate mean and SE.

**B) Expression of RvD1 receptors on sputum leukocytes.** Sputum obtained upon spontaneous expectoration form volunteers with CF was diluted 2-5fold with saline and cells were dispersed (40 sec) using a tissue dissociator (GentleMACS, Miltenyi Biotec, Calderara di Reno, Italy). Leukocytes were counted and stained with VioBlue anti CD45 (clone 5B1), PE-Vio700 anti-CD14 (clone TÜK4), and PerCP-Cy5.5 CD11b (clone M1/70) antibodies (from Miltenyi Biotec), APC anti-ALX/FPR2 (clone 304405, R&D Systems, Minneapolis) and DRV1/GPR32 (GTX71225, GeneTex, Irvine, CA) followed by a PE-labeled secondary antibody, prior to acquisition on a flow cytometer. Shown here are representative dot-plots and histograms from each study participants

**C) Representative histogram plots of flow cytometry results showing increase in blood PMN-associated pHRodo fluorescence.** For each sample a minimum of 3,000 cells in the PMN gate (see inserted FSC/SSC dot plot as an example of gating strategy) were acquired. As a control of pHRodo autofluorescence outside lysosomes, cells kept at 4 °C were included in each experiment to assess baseline fluorescence.

**Supplementary figure 2**

**RvD1 stimulates antimicrobial responses by CF MΦ and reduces *P. aeruginosa* growth on bronchial epithelial cells. A.** Enhancement of *P. aeruginosa* phagocytosis by human CF monocyte-derived MΦ. Results are mean ± SE from experiments with cells from 4 different study participants. *, P < 0.05 *vs* cells with *P. aeruginosa* plus vehicle. **B.** Growth of *P. aeruginosa* biofilm (green) on apical surfaces of fully differentiated CFBEC grown on Transwell supports and infected with GFP-P. *aeruginosa* (1x10^7^ CFU,1 hour). Planktonic bacteria were removed, and cells were treated with vehicle or RvD1 (10 nM) for 3 h. Biofilms were lysed with 0.1 % Triton X-100/PBS and numbers of viable CFU were counted upon spreading on agar plates. Results are mean ± SE from 3 separate experiments, *, P= 0.032 (Student’s *t* test). Confocal microscopy image shows biofilm of GFP-PAO1 growth on CFBEC. Actin staining is shown in red.

Supplementary Table 1. Scale ranging for semiquantitative histopathology assessment of lung sections

|  | **Scale ranging** | | | |
| --- | --- | --- | --- | --- |
| **Parameter** | **0** | **1** | **2** | **3** |
| *Parenchymal involvement* | NONE | 5-10% of parenchyma | 20-40% of parenchyma | >40% of parenchyma |
| *Airway inflammation* | NONE | 1or 2 airways | 3 or 4 airways | > 5 airways |
| *Granulocyte Infiltrate* | NONE | Intraluminal  granulocytes | Intraluminal and interstitial granulocytes | Intraluminal, interstitial and alveolar granulocytes |
| *Lymphocyte infiltrate* | NONE | 1 aggregate/ lung section | 2 aggregates/ lung section | 3 or more /lung section |

Supplementary Table 2

| **Symbol** | **Entrez Gene Name** | **Exp. Fold**  **Change** | **Type(s)** | **Entrez Gene ID Human** |
| --- | --- | --- | --- | --- |
| ABCA1 | ATP binding cassette subfamily A member 1 | -1.14 | transporter | 19 |
| ALOX5AP | arachidonate 5-lipoxygenase activating protein | -1.284 | other | 241 |
| ANGPT1 | angiopoietin 1 | 0.451 | growth factor | 284 |
| AURKB | aurora kinase B | 1.811 | kinase | 9212 |
| BCCIP | BRCA2 and CDKN1A interacting protein | 1.701 | other | 56647 |
| CCL19 | C-C motif chemokine ligand 19 | 1.427 | cytokine | 6363 |
| CCL5 | C-C motif chemokine ligand 5 | -1.410 | cytokine | 6352 |
| CCR5 | C-C motif chemokine receptor 5 | -1.996 | G-protein coupled receptor | 1234 |
| CCRL2 | C-C motif chemokine receptor like 2 | -1.558 | G-protein coupled receptor | 9034 |
| CD14 | CD14 molecule | -1.453 | transmembrane receptor | 929 |
| CD40 | CD40 molecule | -1.091 | transmembrane receptor | 958 |
| CD74 | CD74 molecule | -1.293 | transmembrane receptor | 972 |
| CD80 | CD80 molecule | -1.776 | transmembrane receptor | 941 |
| CD93 | CD93 molecule | 1.742 | other | 22918 |
| CMKLR1 | chemerin chemokine-like receptor 1 | 1.985 | G-protein coupled receptor | 1240 |
| COL4A3BP | collagen type IV alpha 3 binding protein | -0.908 | kinase | 10087 |
| CX3CL1 | C-X3-C motif chemokine ligand 1 | 0.701 | cytokine | 6376 |
| CXCL1 | C-X-C motif chemokine ligand 1 | -1.496 | cytokine | 2919 |
| CXCL8 | C-X-C motif chemokine ligand 8 | -1.343 | cytokine | 3576 |
| DBN1 | drebrin 1 | -1.125 | other | 1627 |
| GLRA2 | glycine receptor alpha 2 | 1.608 | ion channel | 2742 |
| GLRX | glutaredoxin | -1.609 | enzyme | 2745 |
| ICOS | inducible T cell costimulator | -1.555 | transmembrane receptor | 29851 |
| IL10RA | interleukin 10 receptor subunit alpha | 1.73 | transmembrane receptor | 3587 |
| IL18R1 | interleukin 18 receptor 1 | 1.539 | transmembrane receptor | 8809 |
| IL6ST | interleukin 6 signal transducer | -1.336 | transmembrane receptor | 3572 |
| Integrin | -- | NA | complex |  |
| ITGA5 | integrin subunit alpha 5 | 1.479 | transmembrane receptor | 3678 |
| ITGAX | integrin subunit alpha X | -1.43 | transmembrane receptor | 3687 |
| ITPKA | inositol-trisphosphate 3-kinase A | 0.884 | kinase | 3706 |
| KL | klotho | 1.296 | enzyme | 9365 |
| KNG1 | kininogen 1 | -2.005 | other | 3827 |
| KNG1 | kininogen 1 | -1.9365 | other | 3827 |
| LILRB4 | leukocyte immunoglobulin like receptor B4 | -1.05 | other | 11006 |
| LIN7C | lin-7 homolog C, crumbs cell polarity complex component | -1.2 | other | 55327 |
| LTF | lactotransferrin | -1.307 | peptidase | 4057 |
| MED6 | mediator complex subunit 6 | -1.3565 | transcription regulator | 10001 |
| MIF | macrophage migration inhibitory factor | -1.626 | cytokine | 4282 |
| NDUFS6 | NADH:ubiquinone oxidoreductase subunit S6 | -1.201 | enzyme | 4726 |
| NOS3 | nitric oxide synthase 3 | 1.313 | enzyme | 4846 |
| NTRK2 | neurotrophic receptor tyrosine kinase 2 | 0.388 | kinase | 4915 |
| OPRM1 | opioid receptor mu 1 | 0.776 | G-protein coupled receptor | 4988 |
| PEAR1 | platelet endothelial aggregation receptor 1 | 1.026 | other | 375033 |
| PGAP3 | post-GPI attachment to proteins 3 | -1.05 | enzyme | 93210 |
| PLAUR | plasminogen activator, urokinase receptor | -1.309 | transmembrane receptor | 5329 |
| PLG | plasminogen | -1.14 | peptidase | 5340 |
| PPM1B | protein phosphatase, Mg2+/Mn2+ dependent 1B | -0.855 | phosphatase | 5495 |
| PRKCB | protein kinase C beta | 1.931 | kinase | 5579 |
| PSMB6 | proteasome subunit beta 6 | -0.637 | peptidase | 5694 |
| PTGDS | prostaglandin D2 synthase | -1.674 | enzyme | 5730 |
| PTGER2 | prostaglandin E receptor 2 | -1.473 | G-protein coupled receptor | 5732 |
| PTGER4 | prostaglandin E receptor 4 | -1.702 | G-protein coupled receptor | 5734 |
| S100A8 | S100 calcium binding protein A8 | -1.179 | other | 6279 |
| SYK | spleen associated tyrosine kinase | -1.388 | kinase | 6850 |
| TGM2 | transglutaminase 2 | 1.043 | enzyme | 7052 |
| TP63 | tumor protein p63 | 1.811 | transcription regulator | 8626 |
| TREM2 | triggering receptor expressed on myeloid cells 2 | -1.881 | transmembrane receptor | 54209 |
| TRIM31 | tripartite motif containing 31 | -1.704 | other | 11074 |
| TRIM32 | tripartite motif containing 32 | -1.27 | transcription regulator | 22954 |
| TYROBP | TYRO protein tyrosine kinase binding protein | -1.353 | transmembrane receptor | 7305 |
| WNT1 | Wnt family member 1 | 1.925 | cytokine | 7471 |
| WNT7B | Wnt family member 7B | 1.107 | other | 7477 |
