# Supplementary figures and images for: Resolvin D1 Reduces Lung Infection and Inflammation Activating Resolution in Cystic Fibrosis
Source: Front Immunol. 2020 Apr 28;11:581. doi: 10.3389/fimmu.2020.00581 (PMC7247852; doi:10.3389/fimmu.2020.00581)

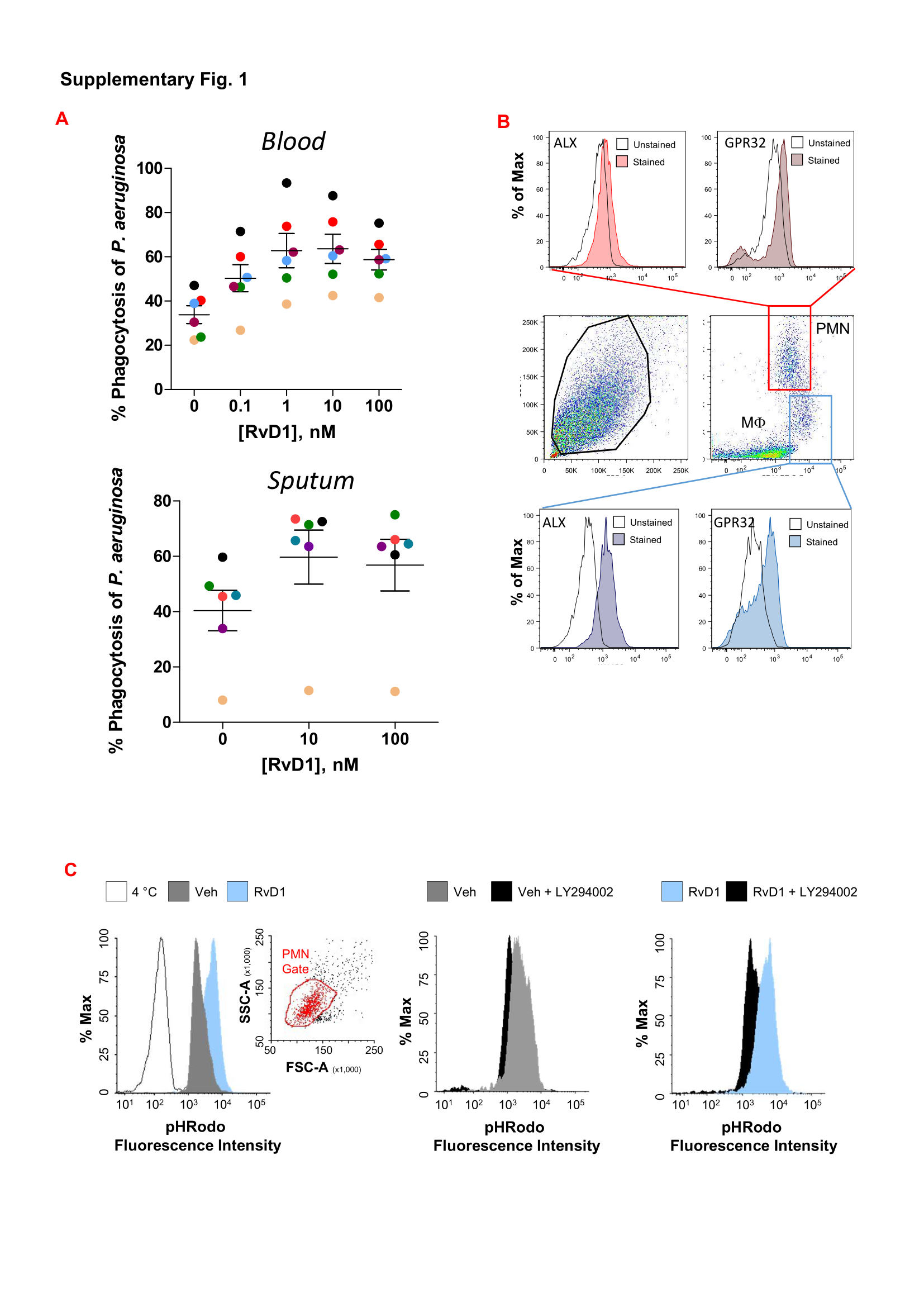

Supplement: Supplementary file 2 [file Image_1.TIFF]

Supplementary Fig. 2

A

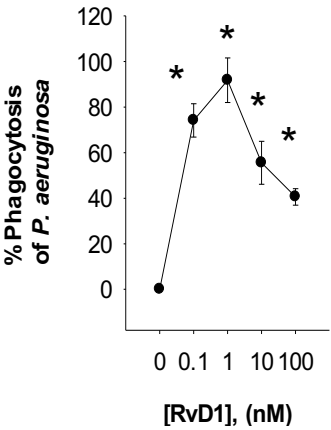

B

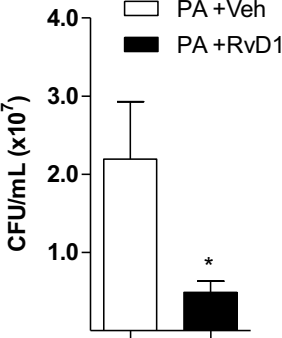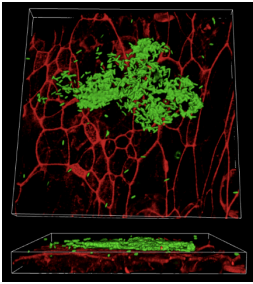

Supplement: Supplementary file 3 [file Image_2.pdf]
